# In Vitro Performance Analysis of a Minoxidil Thermosensitive Gel with Reduced Runoff for Eyebrow Hair Growth

**DOI:** 10.3390/gels9040269

**Published:** 2023-03-24

**Authors:** Luciano C. G. Xavier, Breno N. Matos, Geisa N. Barbalho, Manuel A. Falcão, Marcilio Cunha-Filho, Guilherme M. Gelfuso, Tais Gratieri

**Affiliations:** Laboratory of Food, Drugs, and Cosmetics (LTMAC), University of Brasilia, Brasília 70910-900, Brazil

**Keywords:** minoxidil, hair loss, lateral diffusion, vertical skin penetration

## Abstract

There is a growing interest in innovative products for eyebrow hair loss treatment with fewer adverse effects. Nevertheless, a fundamental formulation aspect of preventing the fragile skin from the ocular region from being irritated is that the formulations remain restricted to the application region and do not run off. Consequently, the methods and protocols in drug delivery scientific research must be adapted to fulfill such performance analysis demand. Thus, this work aimed to propose a novel protocol to evaluate the in vitro performance of a topical gel formulation with a reduced runoff for minoxidil (MXS) delivery to eyebrows. MXS was formulated with 16% poloxamer 407 (PLX) and 0.4% of hydroxypropyl methylcellulose (HPMC). The sol/gel transition temperature, viscosity at 25 °C, and formulation runoff distance on the skin were evaluated to characterize the formulation. The release profile and skin permeation were evaluated in Franz vertical diffusion cells for 12 h and compared to a control formulation (4% PLX and 0.7% HPMC). Then, the formulation’s performance at promoting minoxidil skin penetration with minimum runoff was evaluated in a vertical custom-made permeation template (divided into three areas: superior, middle, and inferior). The MXS release profile from the test formulation was comparable to that from the MXS solution and the control formulation. There was also no difference in the MXS amount that penetrated the skin in the permeation experiments in Franz diffusion cells using the different formulations (*p* > 0.05). However, the test formulation demonstrated a localized MXS delivery at the application site in the vertical permeation experiment. In conclusion, the proposed protocol could differentiate the test formulation from the control, attesting to its better performance in efficiently delivering MXS to the site of interest (middle third of application). The vertical protocol can be easily employed to evaluate other gels with a drip-free appeal.

## 1. Introduction

The eyebrows are structures with protection functions (e.g., protecting the eye from sweat) and have meaning in social and sexual communication, affecting emotions and the interpretation of expressions [1]. Furthermore, eyebrows interfere with facial symmetry (color, shape, density, and hair distribution) and are necessary for facial recognition [2,3].

Hair loss or thinning is an ordinary condition, representing one of the most common diagnoses by the Brazilian Society of Dermatology [4]. Data regarding the percentage of people specifically suffering from eyebrow hair loss are not precise and are difficult to obtain, which adds to the underestimation of the problem. Yet, eyebrow loss is often reported as an additional symptom of numerous diseases as atopic dermatitis [5,6], seborrheic dermatitis [7], hypothyroidism [8], infectious diseases as Hansen disease and syphilis [9], and several autoimmune conditions [10], such as discoid lupus erythematous [11] and scleroderma [12]. However, from all of the autoimmune conditions, alopecia areata stands out for the high number of people affected, with an estimated 2% of the world population being affected at least once in their lives [13]. Even though the aesthetic and social problems of eyebrow hair loss are recognized, specific treatments for eyebrows are still absent [14]. Currently, patients commonly use topically commercial minoxidil hydro-ethanolic solutions. In addition to the limitations of these formulations to deliver high drug doses when applied to dry skin, because of the low drug solubility and crystallization at the skin surface, they can generate adverse effects such as dryness, irritation, burning, redness, and growth of hair in unwanted places [15,16,17]. Consequently, there is a significant demand for products that are free of organic solvents, easy to manipulate, and can attach to the eyebrows for proper delivery. The removal of the formulation is another critical factor inherent to the treatment, as it is essential to have minimal residues and not leave the skin looking oily. Water-soluble and non-irritating polymers are interesting as they allow discomfort-free washing after daily treatment.

Poloxamer (PLX) has been used for developing gels due to its thermoresponsive characteristic, which can be easily applied as a solution and transition into a gel when spread on the skin [9,10]. This polymer has been proposed in several different formulations for application in sensitive tissues or areas such as the eye and burned skin, proving to be non-toxic [16,18]. At a first glance, a thermoresponsive formulation provide the advantage of being easier to apply than a conventional semisolid formulation, i.e., as it is applied as a solution drop, it is easier to spread and get in direct contact with the skin than a conventional formulation. However, more than that, another essential characteristic of an ideal eyebrow formulation is reduced running off, preventing the formulation from reaching undesirable eye regions. For this, other polymers can be added. For example, hydroxypropyl methylcellulose (HPMC) has adhesive and bioadhesive characteristics, which may enhance the formulation viscosity, providing more resistance to deformation [19,20,21,22], and, consequently, reducing runoff.

Nonetheless, a fundamental aspect of the formulation intended for an eyebrow application is not to run off. Conventional in vitro permeation experiments using Franz diffusion cells represent an essential method for formulation screening, evidencing the total amount of drug that penetrates and permeates through the skin and providing data to evaluate the drug distribution to the different skin layers. The best formulations may be selected for further development based on the permeated amount or specific distribution to a target skin layer. However, such a protocol does not differentiate formulations regarding their mechanical performance. When such an aspect is critical for the safety and efficacy of the treatment, other performance analyses must follow.

Based on the above, this work proposes a new modified vertical diffusion skin model to simulate the eyebrow inclination for in vitro permeation experiments allowing reliable in vitro performance analysis of the formulations. Furthermore, a gel formulation containing MXS without organic solvent and reduced runoff to treat eyebrow hair loss was tested and compared to a control formulation. In addition, formulations were characterized regarding their rheological aspects.

## 2. Results and Discussion

### 2.1. Gel Characterization

Thermosensitive copolymers used as in situ forming gels are an exciting strategy to increase the formulation’s residence time at the eyebrows and reduce runoff. Runoff was taken as the distance traveled by a formulation drop on the skin and analyzed according to the polymer concentration. 

PLX was chosen based on its characteristic of T_sol/gel_, as the copolymer can jellify in situ as the temperature increases, while HPMC can provide improvements in the formulation’s mechanical characteristics [19,20]. The rheological behavior of the formulation is critical for droplet dosing, transition temperatures, drug-delivered doses, and application comfort, consequently affecting the treatment efficacy and patient compliance [20]. Regarding the application convenience to the eyebrow region, a liquid formulation would be desirable as it could be applied as a drop [23].

For a formulation to be applied as a drop at room temperature, the T_sol/gel_ must be lower than the temperature of the skin surface (32 °C) [20]. Table 1 shows that the formulation consisting of PLX 16% HPMC 0.4% fulfils this requirement.

The initial assessment time was based on the gelling time, approximately 31 ± 3 s, for the formulation of PLX 16% HPMC 0.4% [24] (Table 1). The runoff in the first minute following the formulation application is a direct measure of the formulation chance to leak and to get in contact with the eye and other undesired areas. Yet, these initial seconds between the administration and the full sol/gel transition are important for the spreading of the formulation. When the formulation is applied as a solution, it has a better contact with the skin and the adjacencies of the hair follicle, hence the drug permeation is favored. Indeed, as stated before, that is one of the advantages of having a thermoresponsive formulation compared to a semisolid conventional formulation. Even though the formulation is intended to treat eyebrow hair loss, when the therapy is initiated there are commonly still hairs present. If the formulation is already very viscous, it may be attached to the hair and not get in direct contact with the skin, which could hinder the drug penetration. For this, we believe 30 s is a good time as it allows for intimate skin contact of the formulation before the full gelation occurs.

The rheological behavior of the formulation is also critical for droplet dosing, transition temperature, drug-delivered dose, and application comfort, consequently affecting the treatment efficacy and patient compliance [25]. 

### 2.2. In Vitro Release Studies

**Figure 1:**
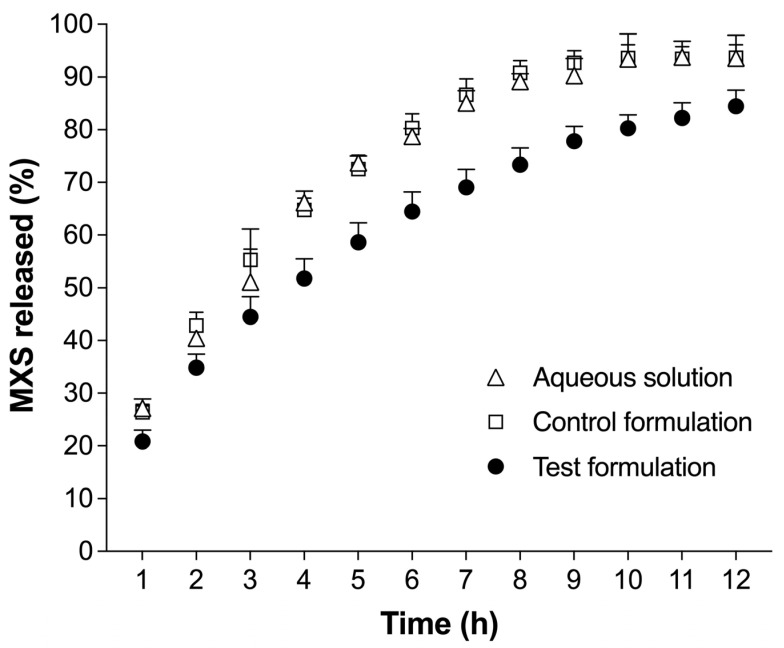
Release profile from test formulation (PLX 16% HPMC 0.4%), control formulation (PLX 4% HPMC 0.7%), and aqueous solution, all containing MXS 2%. Data were expressed as mean ± standard deviation (SD) of 6 replicates for each formulation (*p* > 0.05).

The release study was carried out to evaluate the profile of the MXS release from the test formulation compared to the control formulation and an aqueous control solution with 2% MXS. The result is presented in Figure 1 as the cumulative percentage of the drug recovered in the receptor chamber in relation to the amount applied to the donor chamber. The test and control formulations showed no statistical differences compared to the solution control (*p* > 0.05). Given the results, all MXS is released from the controls after 8 h of experiment, which occurs after 12 h when using the test formulation. Hence, the formulation is not controlling the drug release, which is a positive aspect, as the drug is readily available to penetrate the skin and the hair follicle. When the topical formulation does not control the release, the adjustment of the interval between administration is important to maintain therapeutic concentrations in the tissue. This study may suggest an application of the formulation twice a day, which is suitable for this type of use. The release profile of both the formulations and drug aqueous solution fitted the Higuchi kinetics model (control formulation—R^2^: 0.99; aqueous solution—R^2^: 0.99; and test formulation—R^2^: 0.93). 

### 2.3. Skin Permeation Study in Franz Diffusion Cells

**Figure 2:**
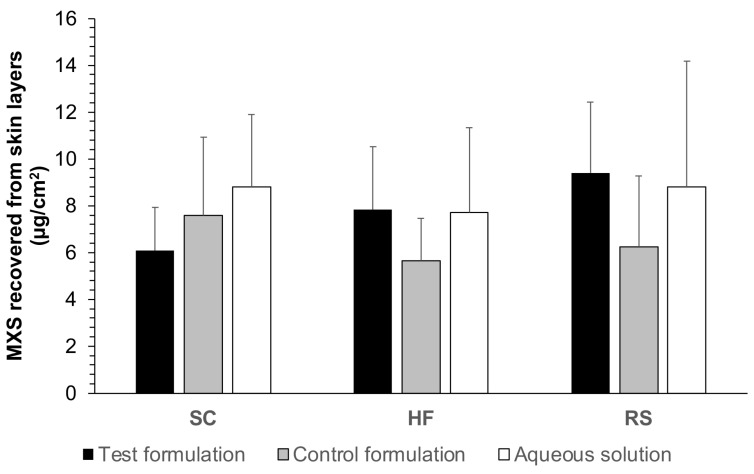
Amount penetrated in µg/cm^2^ of MXS in intact skin after 12 h from test formulation (PLX 16% HPMC 0.4%), control (PLX 4% HPMC 0.7%), and aqueous solution, all containing MXS 2%. SC, HF, and RS. Statistical analysis using two-way ANOVA with multiple comparisons (*p* < 0.05). Data were expressed as mean ± SD of 6 replicates for each formulation.

The results of the in vitro skin permeation study in Franz diffusion cells, i.e., the permeation in which the formulations were applied on the skin horizontally positioned between the donor and receptor compartments of the diffusion cells, are represented in Figure 2.

There was no detection of MXS in the receptor fluid, demonstrating that the formulation would only have a topical action, in which the drug is unlikely to be absorbed systemically, whatever the formulation tested.

There was no statistical difference (*p* > 0.05) between the MXS recovered from the different skin layers from the test, control formulation, and aqueous solution, indicating that there was no decrease in penetration due to the increase in viscosity at the temperature of the experiment (32 ± 2 °C).

This set of experiments could show that following the application of either the test formulation or the controls, the MXS diffused all skin layers homogeneously (stratum corneum, hair follicles, and remaining skin). Hence, the drug distribution profile, in this case, is not a significant screening parameter for selecting a better formulation. 

### 2.4. Vertical Permeation

Permeation studies using the modified Franz cell using porcine ear skin are well-known for comparing formulations [26,27,28]. However, this experiment could not distinguish the test formulation from the control since the crucial difference is the reduced runoff characteristic. As this formulation was designed to be applied to the eyebrows, such a model could not evaluate the formulation’s performance in a vertical situation. Such a vertical situation must not be confused with the concept of lateral diffusion that occurs independent from the skin position. The ability of drugs to diffuse in all directions (vertically and horizontally) is known but still little investigated. Such a lateral diffusion can occur on the surface of the stratum corneum, along the lipid bilayer between the various layers, lipolytic channels, and the corneocytes. In this study, a vertical arrangement was designed to evidence the formulation performance and better understand the product’s actual behavior in a real vertical positioning situation. For this, the lateral diffusion was also quantified and deducted from the results attained as a consequence of the formulation runoff.

Based on the proposed study design, a formulation with a reduced runoff is expected to have a minimum MXS amount in the inferior skin part. However, the lateral diffusion may not be neglected [29,30,31]. For this, the MXS was also quantified in the superior region. Thus, the amount of drug in the superior region can be considered as the amount penetrated through lateral diffusion. The drug amount that penetrated the inferior region as a direct consequence of the contact with the gel that leaked from the application site can be taken as the amount found in the inferior part after subtracting the amount found in the superior part, subtracting, in this way, the MXS reaching this area from a lateral diffusion pathway.

**Figure 3:**
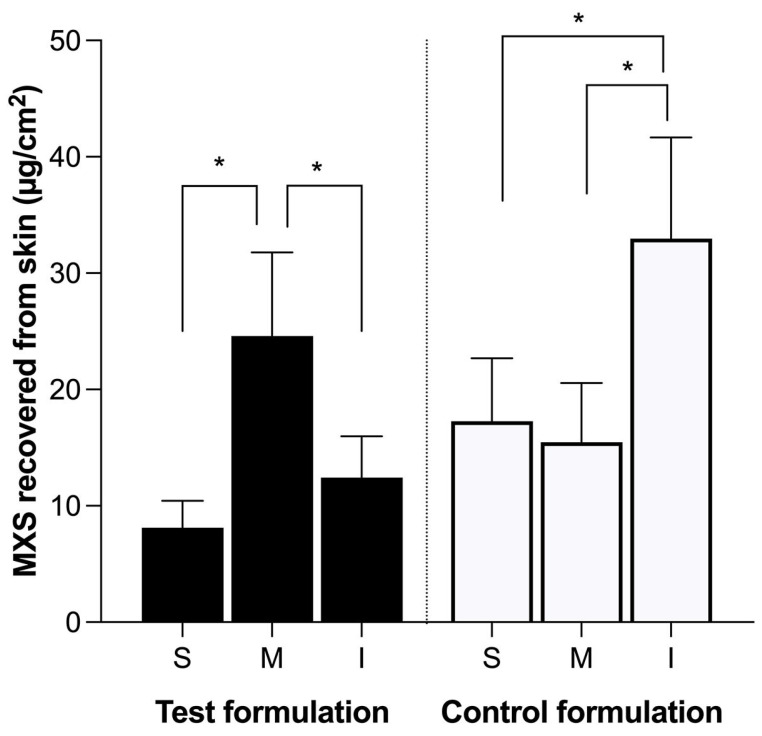
Amount penetrated in µg/cm^2^ of MXS in total skin in the vertical penetration test after 12 h from test formulation (PLX 16% HPMC 0.4%) and control formulation (PLX 4% HPMC 0.7%), both containing 2% MXS. Portions of the total skin represented by: S: superior; M: medium; I: inferior. Statistical analysis using two-way ANOVA with multiple comparisons (*p* < 0.05). * = significant difference. Data were expressed as mean ± SD of 6 replicates for each formulation.

The results for the test formulation compared to the control formulation are shown in Figure 3. The aqueous solution control was not used as its fast draining is already presupposed.

Following the test formulation application, there was no statistical difference in the MXS amount in the inferior and superior parts of the skin (*p* > 0.05) (Figure 3), demonstrating that the MXS present in the inferior area is most certainly a consequence of lateral drug diffusion. Furthermore, drug penetration amounts into the middle section correlate with those found in the Franz diffusion cell permeation test, demonstrating no considerable loss of MXS due to runoff. In contrast to the behavior of the test formulation, the control formulation, which showed a high runoff (Table 1) and low viscosity, provided a higher concentration of MXS in the inferior area of the skin in relation to the other areas (*p* < 0.05). This result validates the use of the designed experiment to evaluate formulation performance and evidences the importance of such a parameter. The here-proposed protocol opens the possibility to be directly applied in the routine of developing novel formulations as a more straightforward approach, avoiding the laborious unnecessary characterization experiments of formulations that may not provide the necessary performance.

### 2.5. Irritation Test

**Figure 4:**
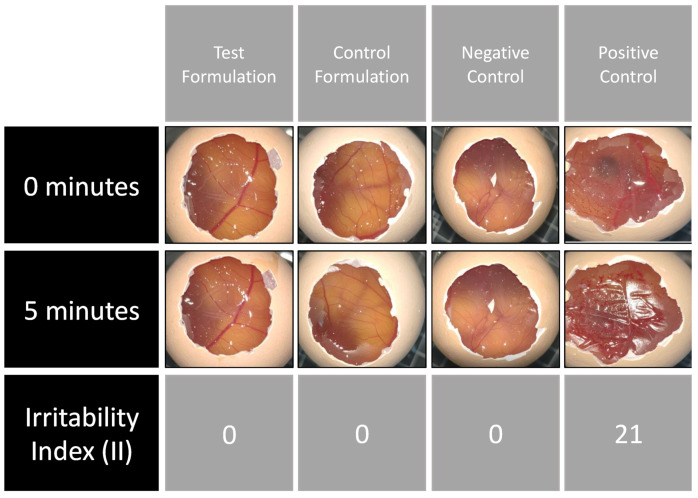
Irritability test (HET-CAM) results, demonstrating the effect of positive (NaOH 1 M) and negative NaCl 0.9%) controls, test and control formulations on the chorioallantoic membrane with 0 min, 5 min, and the Irritability Index (II).

Both the test formulation and control formulation scored zero in the irritability index (II) after 5 min of the chorioallantoic membrane treatment (Figure 4) showed no damage to the blood vessels and no membrane ruptures. HPMC and PLX have already been demonstrated to be non-irritating in other studies [20,32]. Based on the irritability index obtained for both formulations, they can be considered non-irritating and safe for administration. 

## 3. Conclusions

The proposed protocol using a custom-made vertical template could differentiate the test formulation from the control, attesting to its high efficiency in delivering MXS to the site of interest (middle third of application). Furthermore, the method was simple and straightforward, serving as a practical alternative during formulation screening. Instead of performing a complete rheological characterization, which demands sophisticated equipment, an in vitro performance analysis may be used to select the better formulations. Hence, the vertical protocol can be easily employed to evaluate any other topical formulation with a drip-free appeal. 

## 4. Material and Methods

### 4.1. Material

Galena Química Ltda. (Campinas, Brazil) kindly provided the MXS. Affinisol^TM^ (HPMC) and polyethylene glycol 400 (PEG) were obtained from Dynamics (São Paulo, Brazil); poloxamer^®^ 407 (PLX), benzoic acid, and methylene blue dye were obtained by Sigma-Aldrich (Steinheim, Germany). The monobasic and dibasic sodium phosphate were purchased from Vetec (Rio de Janeiro, Brazil), the sodium chloride from Serva (Rio de Janeiro, Brazil), and the sodium hydroxide from Contemporary Chemical Dynamics (Rio de Janeiro, Brazil). The commercial filament acrylonitrile butadiene styrene (ABS) natural for the 3D printing of 1.75 mm was obtained by 3Dfila (Belo Horizonte, Brazil). The modified Saarbruecken cells were printed on the Voolt 3D model Gi3 printer (São Paulo, Brazil), graphically drawn, and sliced with the free versions of Tinkercad^®^ (Autodesk^®^ Inc, San Rafael, CA, USA) and Slic3r^®^ (Rome, Italy) softwares, respectively. The cyanoacrylate glue was obtained from Loctite (São Paulo, Brazil), and the cellulose acetate membrane (MW 12,000 to 4000 Da) was obtained from Fisherbrand (Leicestershire, UK). The HPLC-grade solvents such as methanol and acetonitrile were obtained from JT Baker (Philipsburg, PA, USA). All experiments were carried out with ultrapure water (Milli-Q^®^, Millipore, Illkirch-Graffenstaden, France). Via Carnes (Formosa, Brazil) provided the skin of porcine ears used in skin permeation studies, and Avifran (Planaltina, Brazil) provided the fertilized eggs for the irritability tests.

### 4.2. Formulation Preparation 

The test formulation (TF) was composed of 16% PLX and 0.4% HPMC, and compared to a control formulation composed of 4% PLX and 0.7% HPMC. Both formulations contained benzoic acid (0.05% *w*/*w*), propylene glycol (5.0% *w*/*w*), and minoxidil sulfate (2% *w*/*w*), prepared by solubilization in water under magnetic stirring. PLX was added to the solution and stored in a refrigerator (2–8 °C) for 12 h. HPMC was added and solubilized under magnetic stirring and stored in a refrigerator (2–8 °C) for at least 24 h. 

### 4.3. Rheological Analyses

Formulations’ rheological analyses were evaluated using a Cari-Med DHR-2 rheometer from TA Instruments with cone-plate geometry (40 mm in diameter and first inclination in relation to the plate with a gap of 25 μm). To ensure minimal shear and allow for rest time, 5 min were programmed before each determination.

According to previously published protocols, the tests were performed using a “Peltier” device [20]. The value of the linear viscoelasticity (RVL) region was used in the solution/gel transition temperature (T_sol/gel_) and viscosity-temperature experiments, where the elastic modulus (G′) remained constant. It was set at 10 × 10^−2^, so the range was <1, ensuring less deformation for any sample within this RLV.

The transition temperature solution/gel (T_sol/gel_) was obtained through a temperature ramp from 15 to 50 °C with an increase of 5 °C/min and frequency ranging from 0.1 to 10 Hz, with a gap of 25 μm between the cone and the plate. The T_sol/gel_ point was determined at the crossing point of module G′ and G″. For viscosity analysis, the constant shear rate was measured with a frequency scan of 10 to 10^−3^ Hz to measure its dynamic viscosity η′.

### 4.4. Runoff Test

The formulation runoff test was performed by measuring the distance traveled by a formulation drop (approximately 20 mg) in porcine ear skin placed at a 45° angle, at 32° C, and at room temperature at 3 different times (1, 5, and 10 min) using 10 replicates using a pachymeter to read the runoff. Methylene blue dye was added to each formulation to aid in visualization.

### 4.5. Analytical Method for MXS Quantification

For the quantification of MXS, high-efficiency liquid chromatography (HPLC) with ultraviolet detection (UV) was performed, as previously described [15]. All analyses were carried out in a Shimadzu instrument (LC 20-AD), with a reverse phase C_18_ column (150 mm × 4.6 mm) maintained at 40 °C. A mixture of water: acetonitrile (80:20) (*v*/*v*) was used as the mobile phase with a flow rate of 1.0 mL/min^−1^. The samples’ injection volume was 50 μL and the MXS was detected at 285 nm. MXS recovery from the skin samples was previously validated by pipetting 50 μL, 75 μL, and 100 μL of a methanolic MXS solution (100 μg/mL) on the skin layers: stratum corneum (SC), hair follicle (HF), and remaining skin (RS) separated by tape stripping [33]. Then, the solvent was evaporated, the sample diluted to 5 mL with methanol, extracted stirring for 24 h, filtered in hydrophobic filters with a porosity of 0.22 μm, and analyzed by HPLC/UV. Experiments were performed in triplicates for each volume pipetted, resulting in recoveries from 84 to 98% for SC, from 81 to 100% for RS, and from 88 to 92% for HF [33].

### 4.6. In Vitro Release Profile

The in vitro MXS release profile from the test, control formulation, and an aqueous solution of MXS 2% were determined using Franz diffusion cells at 35 ± 2 °C. The donor and receptor chambers were separated by a hydrophilic acetate membrane. In the donor chamber, 1 mL of each formulation was added. The receptor chamber was filled with 15 mL of PBS and kept under 500 rpm magnetic stirring (Sink conditions). Over 12 h of the experiment, 1 mL was collected hourly, and the collected volume was immediately replaced. The obtained data were analyzed to determine the order of release [34]. The collected volumes were analyzed by HPLC/UV.

### 4.7. Skin Permeation Study in Franz Diffusion Cells

Franz’s diffusion cells were mounted with porcine skin separating the donor from the receptor chamber. Like the in vitro release profile experiment, 1 mL of each formulation (test formulation and control) was added to the donor chamber. The receptor chamber was filled with 15 mL of PBS and kept under 500 rpm magnetic stirring. Over 12 h of the experiment, 1 mL was collected every 3 h, and the collected volume was immediately replaced. The collected volumes were analyzed by HPLC/UV.

After 12 h of the experiment, the amount of MXS that remained retained in the layers of the SC, HF, and RS was determined using differential tape-striping, as previously described [35]. Briefly, the porcine skin was removed from the Franz diffusion cells, stretched, and fixated on a Styrofoam holder. Next, the SC was removed with 15 pieces of 3M adhesive tape, applied, and removed from the skin in a single motion. The HF was then collected by applying 2 drops of cyanoacrylate glue on the skin and pressing lightly for 5 min until the glue dwelled. Finally, RS, SC tapes, and HF were cut into small pieces and transferred to a bottle containing 5 mL of methanol. The content was filtered in hydrophobic filters with a porosity of 0.45 μm and analyzed by HPLC/UV.

### 4.8. Vertical Permeation Study

#### 4.8.1. Design and 3D Printing of Vertical Permeation Cells

**Figure 5:**
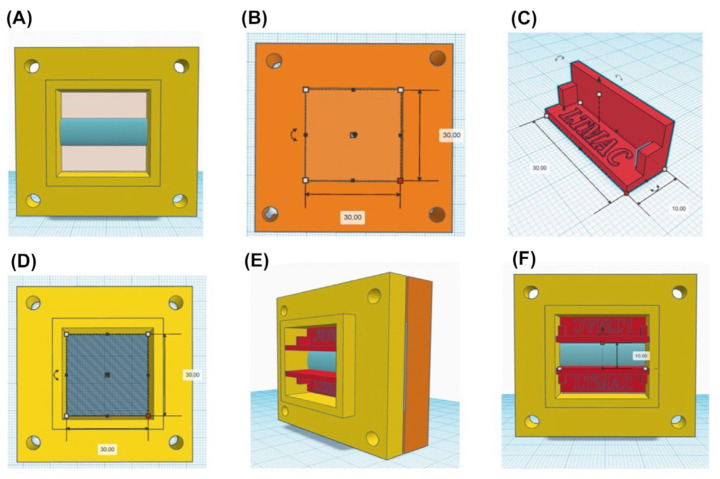
Virtual models of vertical permeation device parts. (**A**) There is an internal area of 9 cm^2^, where the skin is exposed; (**B**) the lower part consists of an internal area of 9 cm^2^ and a depth of 4 mm, which is intended for filter paper; (**C**) templates limit the area of formulation application on the skin; (**D**,**E**) formulation application with the templates; (**F**) representation of the model after template removal.

The vertical permeation cells were developed in a web application for 3D—Tinkercad projects of Autodesk. The parts were printed on the Voolt 3D model Gi3 3D printer based on Voolt 3D’s natural ABS polymer. The developed cell was divided into three parts: top, bottom, and templates, as shown in Figure 5.

#### 4.8.2. Protocol

The study used the custom-made permeation cells described above. For this, the skin was cut in an area of 4 × 4 cm^2^, positioned in the piece’s lower part (Figure 5B), and fixed by the upper part (Figure 1A), with the delimitation of an exposure area of 3 cm^2^ with 2 templates. After the formulation application, both templates were removed.

The assembled cells were previously heated to a temperature of 32 ± 2 °C in an oven. In total, 400 μL of each gel was deposited horizontally, and the skin was immediately positioned at an angle of approximately 15° to avoid upper area contamination. After 5 min of heating, the templates were removed and the cells were positioned vertically for 12 h. After this period, the skin was removed from the cells and washed until the gel was removed. Excess water was removed with paper tissues.

**Figure 6:**
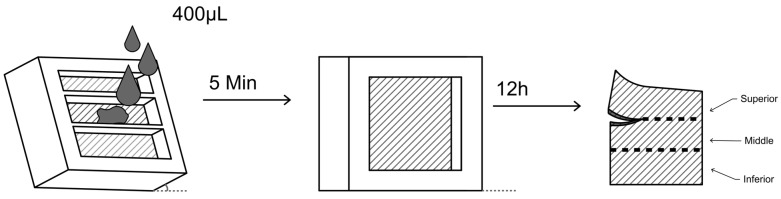
Vertical permeation study schematic rationale, with the formulation application, permeation, and skin division.

The skin prepared for MXS extraction was divided into 3 equal parts of 3 cm^2^ (Figure 6), differentiating the superior, middle, and inferior parts. This division was made to evaluate the lateral diffusion of MXS. The total skin was cut into small pieces, placed in vials with 5 mL of methanol, and magnetically stirred at 500 rpm for 24 h for drug extraction. The content was filtered and analyzed by HPLC/UV.

### 4.9. Irritation Test

The formulations levels of irritancy were assessed using hen’s eggs on the 10th day of fertilization (HET-CAM). Each eggshell was opened at the side of the air chamber, then 300 μL of each testing formulation was applied directly on the chorioallantoic membrane surface. After 20 s, the membrane was carefully washed with a saline solution until the complete sample removal. Each chorioallantoic membrane was visually monitored and photographed for 5 min for hyperemia, hemorrhage, or clotting [35,36]. The irritation severity was classified as: 0 = no reaction, 1 = slight/mild, 2 = moderate, and 3 = severe for each time point (0.5, 2, and 5 min), which allowed for the classification of the irritability index (II) for each sample [37]. A NaCl solution at 0.9% (*w*/*v*) and 1 mol/L NaOH were used as negative and positive controls, respectively.

### 4.10. Statistical Analyses

Statistical analysis was performed in all tests with GraphPad Prism 9 (GraphPad Inc., San Diego, CA, USA). The results with parametric behavior were compared using one-way ANOVA followed by Tukey’s post-test. The non-parametric results, on the other hand, were evaluated by the Kruskal-Wallis test, followed by Dunn’s post-test. The significance level (p) was set at 0.05.

## Figures and Tables

**Table 1:** Test and control formulation runoff distance, T_sol/gel_ (°C), and viscosity.

Formulation	Code	Runoff Test			T_sol/gel_ (° C)	Dynamic Viscosity η’ (Pa.s)
		1 min (mm)	5 min (mm)	10 min (mm)		
PLX 16% HPMC 0.4%	Test	7.2 ± 1.1	7.0 ± 1.1	7.5 ± 1.0	30.4 ± 4.5	82.0 ± 10.0
PLX 4% HPMC 0.7%	Control	15.8 ± 2.5	18.8 ± 3.2	18.9 ± 3.6	-	1.6 ± 0.6

## Data Availability

Data might be available upon request.
